# A study on improving the current density performances of CO_2_ electrolysers

**DOI:** 10.1038/s41598-021-90581-0

**Published:** 2021-05-27

**Authors:** Yueyuan Gu, Jucai Wei, Xu Wu, Xiaoteng Liu

**Affiliations:** 1 School of Environmental Science and Engineering, Huazhong University of Science and Technology, Wuhan, 430074 China; 2 Department of Mechanical and Construction Engineering, Faculty of Engineering and Environment, Northumbria University, Newcastle upon Tyne, NE1 8ST UK

**Keywords:** Environmental sciences, Energy science and technology, Engineering

## Abstract

Electrochemical CO_2_ reduction reaction (CO_2_RR) technology can reduce CO_2_ emission with converting excess electrical energy to high-value-added chemicals, which however needs further improvement on the electrolyser cell performance. In this work, extensive factors were explored in continuous CO_2_ electrolysers. Gold, one of the benchmark materials for CO_2_RR to produce CO, was used as the catalyst. Electrolyser configurations and membrane types have significant influences on cell performance. Compact MEA-constructed gas-phase electrolyser showed better catalytic performance and lower energy consumption. The gas diffusion electrode with a 7:1 mass ratio of total-catalyst-to-polytetrafluoroethylene (PTFE) ionomer exhibited the best performance. At a low total cell voltage of 2.2 V, the partial current density of CO production achieved 196.8 mA cm^−2^, with 90.6% current efficiency and 60.4% energy efficiency for CO producing respectively. Higher CO selectivity can be achieved using anion exchange membranes, while higher selectivity for hydrogen and formate products can be achieved with cation exchange membranes. This research has pointed out a way on how to improve the CO_2_RR catalytic performance in flow cells, leaving aside the characteristics of the catalyst itself.

## Introduction

Achieving the peak carbon dioxide emissions and carbon–neutral objective requires advanced CO_2_ utilization technologies and reliable high-capacity renewable energy storage systems, for which the reduction of CO_2_ to produce valuable chemicals by direct electrolysis is a promising approach^1–5^. Currently, one of the main problems of CO_2_ electrolysers is the insufficient time–space yield or current density at working voltages^6,7^.

In addition to the inherent properties of the cathode catalyst, all components of the flow-cell may have impacts on the electrolyser cell performance^8,9^, including but not limited to electrode preparation processes^10,11^, electrolyte compositions and concentrations, temperatures^12,13^, gas pressures^14^, electrochemical membrane reactor configurations^8^, polymer electrolyte membranes^15^, and polymeric binders^16^. Jhong et al*.*^10^ compared the influence of catalyst layer deposition methodology on electrode catalytic performance, the partial current density of CO production (*j*_*CO*_) obtained with the air-brushed cathode was higher (~ 87 mA cm^−2^ at − 1.68 V vs. Ag/AgCl) than that of the hand-painted cathode, with an increase of about 10 mA cm^−2^. Kim et al*.*^17^ conducted a series of studies to explore the effects of the microporous layer (MPL) composition and the carbon paper substrate on CO_2_RR performance. With a suitable MPL composition (20 wt% PTFE, 1 mg cm^-2^ carbon), the *j*_*CO*_ achieved 280 mA cm^−2^ at −2.20 V vs*.* Ag/AgCl, which was ten times that of the gas diffusion electrode (GDE) without MPL. Concerning the impact of the carbon paper substrate thickness, a higher *j*_*CO*_ (171.5 mA cm^−2^, at −2.05 V vs. Ag/AgCl) was obtained with a thinner substrate (190 µm)^17^. Also, Kim et al*.*^17^ found that carbon paper with a lower wet proof level (10%) can obtain a higher *j*_*CO*_ (224 mA cm^−2^ at −2.05 V vs. Ag/AgCl). However, research by Park et al*.*^18^ showed that the superhydrophobic carbon paper performed best, and the *j*_*CO*_ achieved 19.25 mA cm^−2^ at −1.3 V vs. RHE. Of note, the carbon paper characteristics, flow-cell configurations, and operating conditions used in the above two studies were different. There was no unified conclusion on the effect of substrate hydrophobicity on CO_2_RR performance. Lee et al*.*^16^ found that the PTFE binder exhibited the highest current efficiency for CO production among the five kinds of polymeric binders, 94.7% at − 0.7 V vs. RHE. The mass ratio of catalysts-to-binders used in CO_2_RR-related researches varied from 4:1 to 30:1^19,20^, and even no polymeric binders were added sometimes^15^. The amount of polymeric binder added still needs optimization. Beyond the catalyst, there is still much to be done on improving the CO_2_RR performance.

In this paper, extensive attempts have been made to obtain larger current density at lower cell potentials. First, two electrolyser structures and two different cathode feeding methods were tested and compared. The zero-gap MEA structure with low resistance was preferred, and the humidified gas-phase CO_2_ was directly fed into the cathode to alleviate the mass transfer limitation caused by the low solubility of carbon dioxide in an aqueous solution. Then, through the test and comparison of Nafion and PTFE binders, it was found that adding a small amount of PTFE can improve the hydrophobicity of GDE and obtain a higher selectivity in CO production. Besides, the ion transfer mechanism of the anion exchange membrane was more advantageous in terms of catalytic reduction of CO_2_ to CO.

## Results and discussion

### Influence of the electrolyser structures and the cathode feeding method

**Figure 1:**
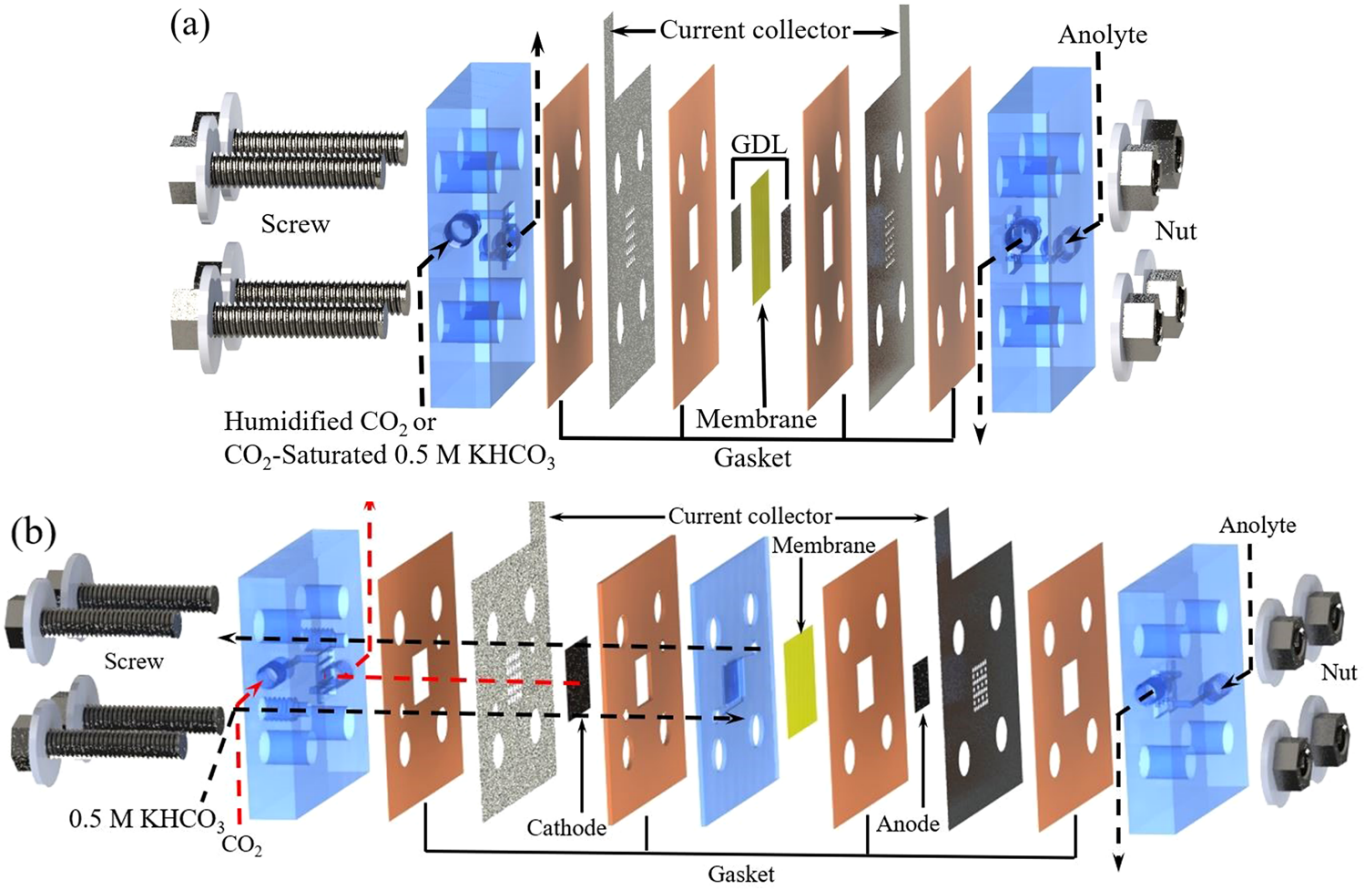
Expanded view of the CO_2_ electrolysers: **(a)** zero-gap MEA configuration, **(b)** with a liquid buffer layer at the cathode side.

CO_2_ electrolysers with two different structures were adopted in this paper, as shown in Fig. 1. Both adopted a common membrane electrode assembly (MEA) configuration^7,21^, except that a liquid buffer layer was added at the cathode side of the second one. Two different cathode feeding methods were used in the MEA configuration (Fig. 1a). For the second one (Fig. 1b), there was a 2 mm thick liquid buffer layer between the membrane and cathode, CO_2_ gas (dry) diffused from the back of the gas diffusion electrode to the catalyst surface.

**Figure 2:**
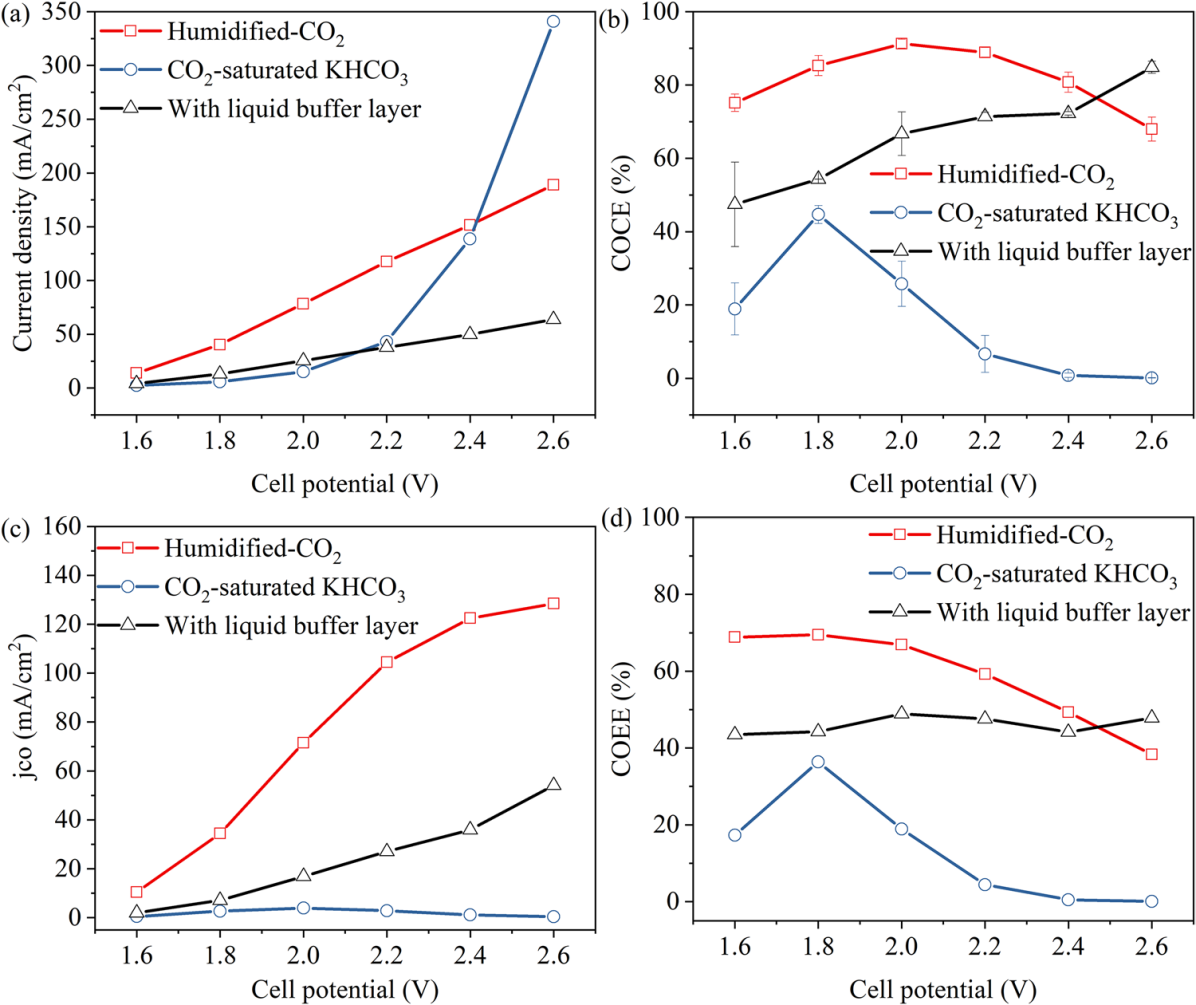
Cell performances acquired with different cathode feeding methods or electrolyser configurations. **(a)** Average total current density, **(b)** COCE, **(c)** average partial current density, and **(d)** COEE, as a function of the total cell potential.

The cell performances of the CO_2_ electrolyser under three different conditions (Figure S3) were shown in Fig. 2. As shown in Fig. 2a, the total current densities obtained with humidified-CO_2_ and liquid buffer layer were almost linearly related to the cell potential (resistance polarization control), and the resistances were 5.6 Ω and 16.6 Ω respectively. The total current density (*j*_*total*_) obtained with CO_2_-saturated KHCO_3_ increased rapidly when the cell potential was higher than 2.2 V, obtained the highest value of 340.9 mA cm^−2^ at 2.6 V. Combined with the product detection results (Fig. 2b), the current increase in this case mainly came from the side reaction of hydrogen evolution. The main products were CO and H_2_, and the current efficiency of H_2_ formation was not indicated in Fig. 2b.

The different cathode feeding methods affected the available CO_2_ concentration on the catalyst surface. As shown in Fig. 2b, when the amount of available reactant gas CO_2_ was sufficient (humidified-CO_2_ feeding method), a current density for CO production (COCE) more than 80% was obtained between 1.8 V and 2.4 V, and the COCE gradually decreased from 2.2 V. Due to the low solubility of carbon dioxide in aqueous solution, there were not that much reactant gases used to produce CO with the CO_2_-saturated KHCO_3_ feeding method. Therefore, the total reduction current density obtained between 1.6 and 2.2 V was much lower than that of the gas-phase feeding method. Until the cell voltage was up to 2.4 V and 2.6 V, the H_2_ evolution selectivity increased, and the total current density increased rapidly. The *j*_*CO*_ was in this order (see Fig. 2c): humidified-CO_2_ > with diffusion layer > CO_2_-saturated KHCO_3_. The corresponding *j*_*CO*_ were 128.4 mA cm^−2^, 54.1 mA cm^−2^ and 0.4 mA cm^–2^ at 2.6 V cell potential, respectively.

By adding a thin liquid pH buffer layer, a triple-phase boundary can be formed. The gas-phase CO_2_ molecules can be quickly diffused to the surface of the catalyst (compared with that in the liquid phase), in this way the CO_2_RR catalytic selectivity can be improved and the hydrogen evolution reaction can be partially suppressed^22^. According to Weekes et al*.*^23^, the mass transfer limitations can be alleviated by using a gas-phase stream, thereby increasing the current density. This can explain why the *j*_*CO*_ obtained with CO_2_-saturated KHCO_3_ was the lowest (less than 4 mA cm^−2^), as the mass transfer of CO_2_ molecules under this condition was the worst^24^. When the cell potential was between 1.6 and 2.2 V, the energy efficiency for producing CO (COEE) obtained with humidified-CO_2_ remained above 60% (see Fig. 2d).

**Figure 3:**
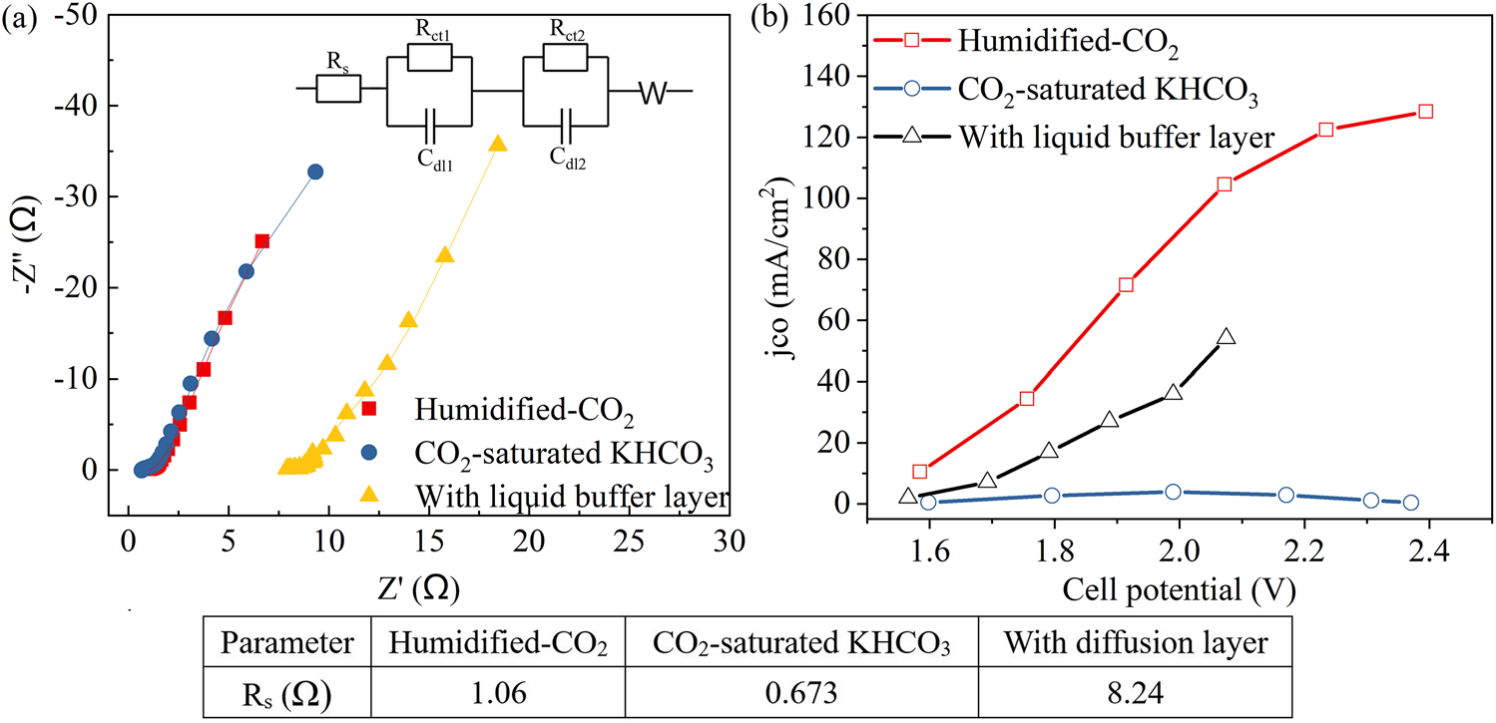
**(a)** EIS results and the relevant equivalent circuits acquired with different cathode feeding methods or configurations under open-circuit level. Points and lines represent measured and fitted results, respectively. **(b)** *j*_*CO*_ as a function of the cell potential (after iR compensation).

As shown in Fig. 3a, the overall resistance between the two electrodes (R_s_) increased significantly (from 1.06 to 8.24 Ω) after adding a liquid buffer layer. This explained the decrease in current density after adding a liquid buffer layer, the large resistance led to the decrease of current density. This also means an increase in energy consumption in industrial applications. The Rs of the electrolyte feeding mode was smaller than that of the gas-phase mode. That is, under the same total cell voltage, the actual potential applied on the cathode with electrolyte feeding mode was slightly higher than that with gas-phase feeding mode, which also has some influence on the production selectivity. The thickness of the buffer layer needs to be extremely thin for better application^8^. As shown in Fig. 3b, after *iR* compensation, the best performance was still obtained with the MEA structure, humidified-CO_2_ feeding method, so this mode was adopted in the following research.

Besides, a small number of hydrocarbons (CH_4_, C_2_H_4,_ and C_2_H_6_) were detected when using CO_2_-saturated KHCO_3_ as a catholyte. As shown in Figure S4, the current efficiency for CH_4_ production was 2.3% at 2.4 V. In contrast, the CE of hydrocarbon product was negligible (less than 0.05%) in the tests under the other two conditions.

### Binder types and contents

#### Cell performance

**Figure 4:**
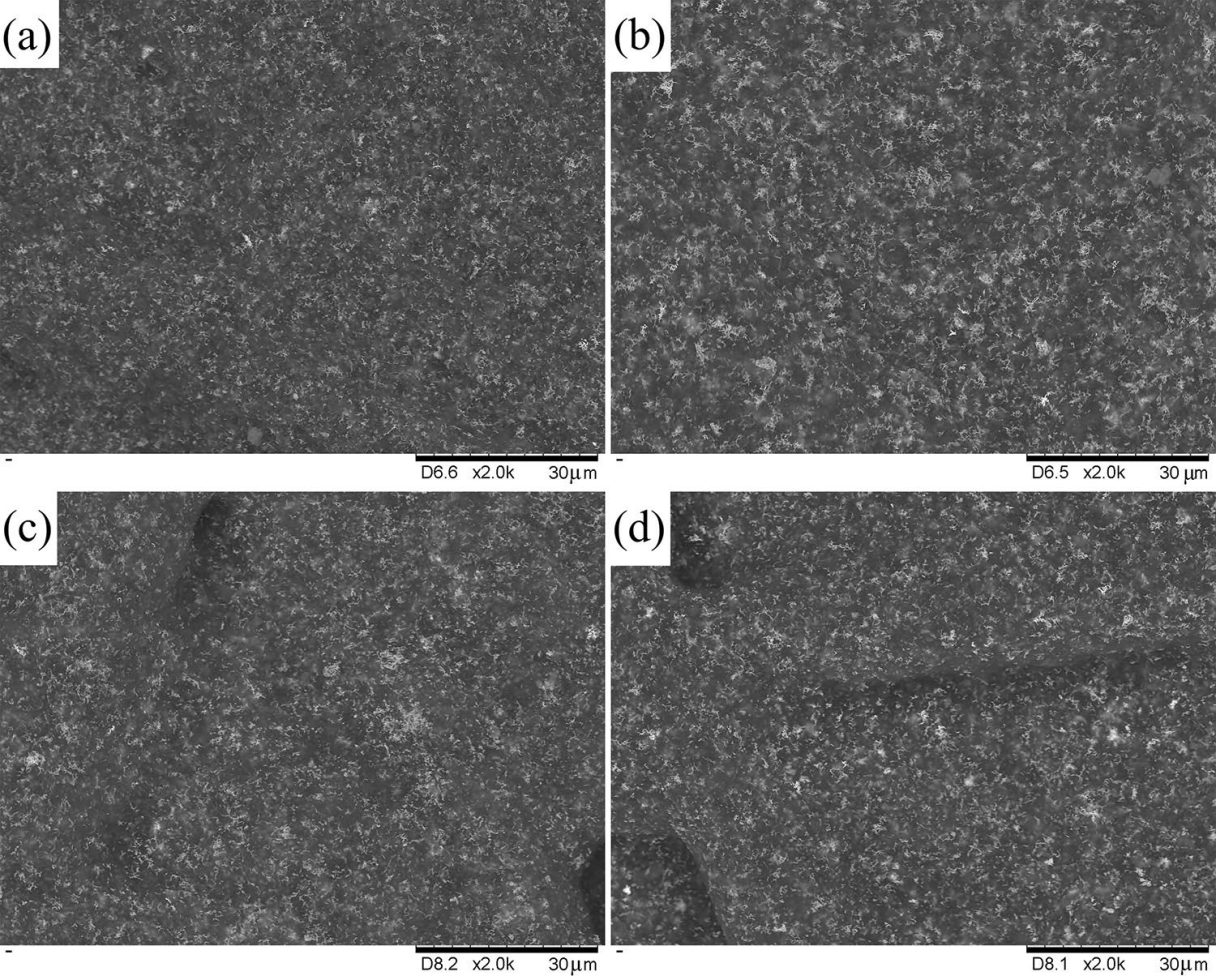
SEM images of top-down views of the gas diffusion electrodes with different Nafion ionomer additions **(a)** 3:1, **(b)** 5:1, **(c) **7:1, **(d)** 10:1.

Two commonly used binders were used to prepare gas diffusion electrodes (GDE). The morphologies of GDE prepared with different Nafion contents were shown in Fig. 4. The catalyst layer was uniformly distributed on the surface of the gas diffusion electrode using the air-brush method. There was no obvious difference in the morphologies of the electrodes prepared with different Nafion contents.

**Figure 5:**
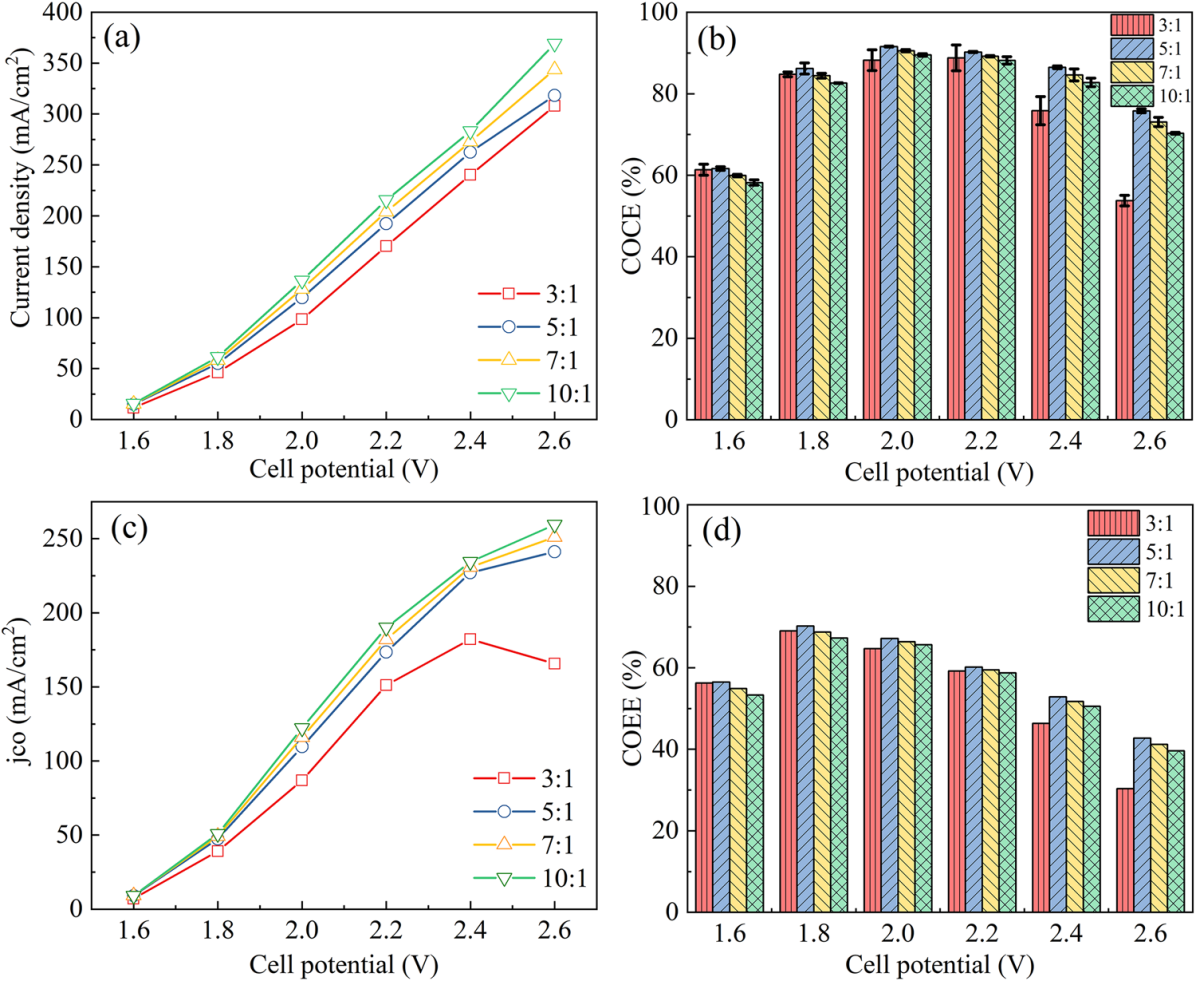
Cell performance of the electrodes with different Nafion contents. **(a)** *j*_*total*_ and **(c)** *j*_*CO*_, **(b)** COCE and **(d)** COEE, as a function of the cell potential.

As shown in Fig. 5a,c, *j*_*total*_ and *j*_*CO*_ were both in this order: 10:1 > 7:1 > 5:1 > 3:1. That is, the more Nafion binder added, the lower the current density. There was no obvious difference between the four samples in Fig. 5b except for the much lower COCEs of the 3:1 sample at high cell potentials (2.4 V and 2.6 V). The range of the four binder ratios was narrow, and the inherent properties of the Au/CN catalyst had a greater impact on catalytic performance, the COCE of the four samples followed the same trend. The *j*_*CO*_ of the 3:1 sample (the highest amount of Nafion added) was much lower than the other three samples, especially at 2.6 V, the *j*_*CO*_ was only 165.7 mA cm^−2^, while the 10:1 sample was 259.5 mA cm^−2^ (with 93.8 mA cm^−2^ difference). This may be ascribed that Nafion is a hydrophilic resin with no hydrophobic gas-phase channels. Especially in high current density zones, adding too much Nafion may make it difficult for the reaction gas CO_2_ to be transported to the catalyst surface^25^. The COEE results were shown in Fig. 5d, and the highest energy efficiency (approximately 70%) was achieved at 1.8 V. When the mass ratio of total-catalyst-to-Nafion was 7:1, the *j*_*CO*_ reached 116.0 mA cm^−2^ at 2.0 V cell potential, the COCE and COEE were 90.6% and 66.4%, respectively.

**Figure 6:**
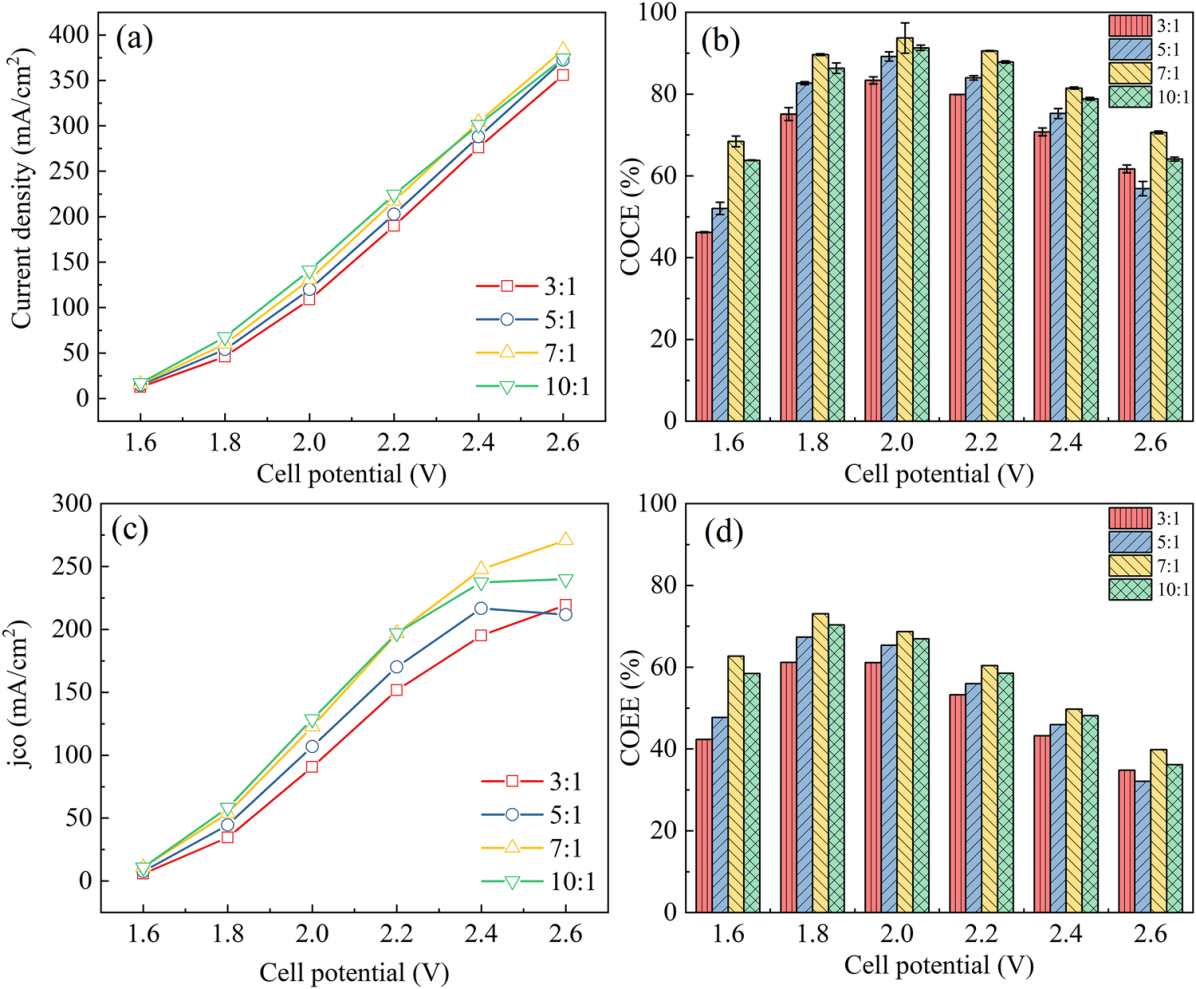
Cell performance of the electrodes with different PTFE contents. **(a)** *j*_*total*_ and **(c)** *j*_*CO*_, **(b)** COCE and **(d)** COEE, as a function of the cell potential.

Similarly, four GDEs with different PTFE binder contents were prepared. The morphologies of GDEs were shown in Figure S5, and there was no obvious difference in appearance. As shown in Fig. 6a, there was not much difference in the total current density of the four electrodes, the total current density of the 3:1 sample was the lowest, similar to the results obtained with the Nafion binder. As the cell potential increased, the COCE of the four electrodes increased first and then decreased, reaching a maximum value (~ 90%) at 2.0 V (see Fig. 6b). Increasing the PTFE addition amount, the COCE of the four electrodes increased first and then decreased, reaching the maximum value when the mass ratio of total-catalyst-to-PTFE was 7:1. Except that at 2.6 V, the COCE of the 3:1 sample was slightly higher than that of 5:1. When the cell voltage was between 1.6 and 2.4 V, the *j*_*CO*_ of the 3:1 sample was lower than that of the 5:1 sample, but at 2.6 V, the *j*_*CO*_ of the 3:1 sample was higher than that of 5:1 (see Fig. 6c). The *j*_*CO*_ of the 7:1 sample reached 122.7 mA cm^−2^ at 2.0 V cell potential, with 93.7% COCE and 68.7% COEE. As shown in Fig. 6d, the maximum energy efficiency (~ 70%) was reached at 1.8 V, which was consistent with the results obtained with Nafion ionomer (see Fig. 5d).

### Electrochemical and hydrophilicity characterization

Too much polymeric binder addition may have a cladding effect on gold particles, which will reduce the active surface area of the gold catalyst. If an Au particle is completely covered by an ionomer, or a C particle loaded with Au particles is covered by an ionomer, it is difficult to conduct electrons with the surrounding C particles. Under such conditions, these Au particles cannot conduct electrochemical reactions, and their surface areas cannot be measured by the CV curves^26^. As shown in Figure S8, the CVs of the electrodes prepared with different binder contents coincide, and the calculated active surface area of gold was about 3.6 cm^2^ (see Table S1). The slight differences in electrochemical surface area (ECSA) may come from weighing errors or pipetting errors. The addition ratios in this research did not have a coating effect on the surface of the gold particles or they are at the same cladding level. 20% to 35% of Nafion polymeric binder addition was generally considered suitable in water electrolysers and fuel cells^27,28^. The highest ratio (3:1) used in this study was included in this optimal range. Therefore, it can be considered that none of these four binder ratios hindered the utilization of gold catalysts.

The Tafel plots were shown in Figure S9, the corresponding Tafel slope and exchange current density (*i*_*0*_) were shown in Table S1. For the Nafion binder, the Tafel slope of the 3:1 sample was significantly higher than the others, while the Tafel slope of the 7:1 sample was the minimum. For PTFE ionomer, the Tafel slope was in this order with a slightly difference:10:1 < 7:1 < 5:1 < 3:1. The lower Tafel slope indicates a faster first-electron transfer step^29^, while higher *i*_*0*_ indicates easier electrode polarization^30^. The electrochemical reduction of CO_2_ to CO on gold under neutral to alkaline pH can be written as follows^31–33^, where * denotes an adsorption site:

1 $${CO}_{2}(\mathrm{g})+{e}^{-}+*\to {*CO}_{2}^{-}$$

2 $${*CO}_{2}^{-}+{\mathrm{H}}_{2}O\to *COOH+{OH}^{-}$$

3 $$*COOH+{e}^{-}\to *{COOH}^{-}$$

4 $$*{COOH}^{-}\to *CO+{OH}^{-}$$

5 $$*CO\to \mathrm{CO}(\mathrm{g})+*$$

If Eqs. (1) or (3) was the rate-determining step, the Tafel slope should be 116 and 39 mV dec^−1^, respectively^34^. The Tafel slope for all electrodes was between 143 to 187 mV dec^−1^, the rate-determining step was closer to Eq. (1).

**Figure 7:**
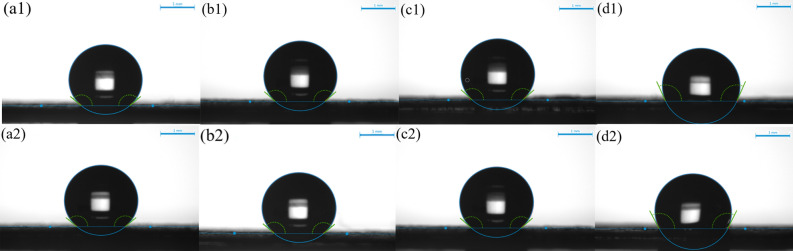
The contact angles of the air-brushed electrodes with different ionomer contents, (1) Nafion and (2) PTFE respectively. **(a)** 3:1, **(b)** 5:1, **(c)** 7:1, **(d)** 10:1.

The static contact angles of water on the gas diffusion electrodes were shown in Fig. 7, and the corresponding specific angle values were shown in Table S1. For both Nafion and PTFE binders, the hydrophobicity of the electrode decreased as the binder content increased. GDEs prepared with PTFE binder were more hydrophobic than those prepared with Nafion binder. The water and gas distribution management of the GDE is important, and it needs to have the functions of gas transporting, liquid discharging, and electrons transferring^35^. Moderate hydrophobicity can improve the management of gas and liquid distribution and decrease the possibility of electrode flooding. Nafion can conduct protons but not electrons, and the increased local proton concentration may promote hydrogen evolution reaction. PTFE can neither conduct electrons nor protons, but it can form hydrophobic pores, which is beneficial to water and gas distribution management^16^. However, adding a large amount of PTFE will increase the internal resistance of the electrode. There should be an optimum value in the balance between conductivity and hydrophobicity. In this study, the best performance was obtained with the 7:1 mass ratio of total-catalyst-to-PTFE.

As shown in Table 1, the CO_2_ electrolysers in this research exhibited excellent cell performance. RT means room temperature, and if no reference electrode was specified, then the potential signified total cell voltage. The mass ratio of catalyst and binder was estimated by the given parameters in each research. In this work, higher partial current density, current efficiency, and energy efficiency in producing CO can be obtained at a lower cell potential, and no heating source was required. The partial current densities for CO production obtained at 2.0 V, 2.2 V and 2.4 V were 122.7 mA cm^−2^, 196.8 mA cm^−2^ and 247.7 mA cm^−2^, respectively. With such a low total cell potential of 2.2 V, a high mass activity for CO production of 985 A/g_Au_ was achieved at room temperature.

**Table 1:** Summary of reported CO_2_ electrolyzers using Au catalyst.

The mass ratio of catalyst and binder	*j*_*CO*_ (mA cm^−2^)	Potential (V)	COCE (%)	COEE (%)	Mass activity for CO (A/g_Au_)	Temperature (^o^C)	Ref
7:1	122.7	2.0	93.8	68.7	613.5	RT	This work
7:1	196.8	2.2	90.6	60.4	984.1	RT	This work
7:1	247.7	2.4	81.5	49.8	1238.5	RT	This work
3:1	~ 148	3.0	88	43	1057	RT	^7^
Not given	> 425	3.0	> 85	> 42	> 1063	60	^13^
8:1	203	2.5	64	38	1128	RT	^34^
30:1	160	− 1.78 V vs. Ag/AgCl	> 60	–	941	RT	^36^
Not given	~ 4	2.0	62	45	231	RT	^37^
None	~ 28	− 1.3 V vs. RHE	77	–	–	RT	^18^

### The influences of membranes

**Figure 8:**
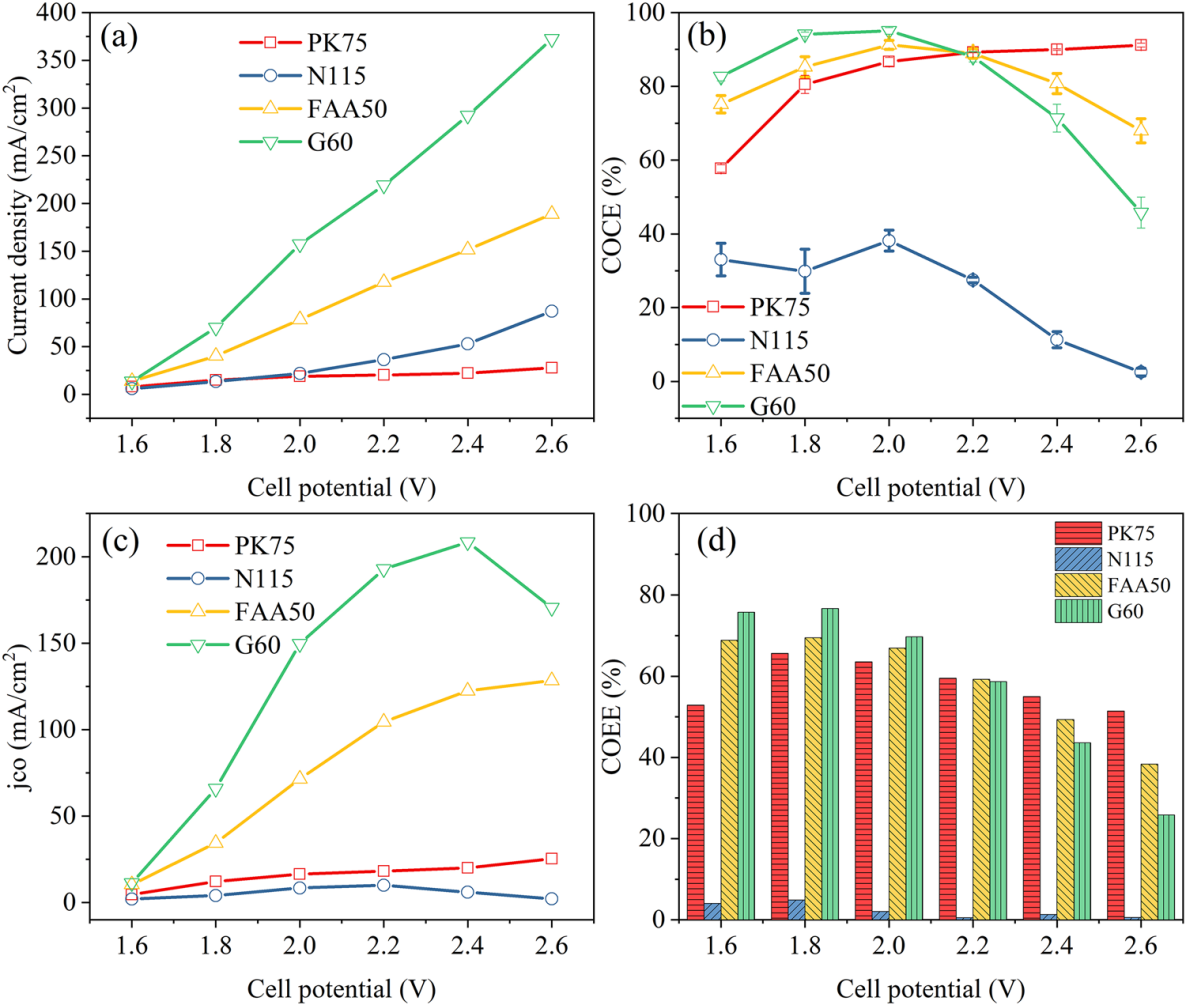
Cell performance acquired with different membrane. **(a)** *j*_*total*_, **(b)** COCE, **(c)** *j*_*CO*_, and **(d)** COEE, as a function of the cell potential.

The CO_2_RR performances with different membranes were shown in Fig. 8, the *j*_*total*_ values were in this order: G60 > FAA50 > N115 > PK75 (see Fig. 8a). The polarization curves of FAA50 and G60 membrane conform to ohmic polarization, and the calculated resistances are 5.6 Ω and 2.8 Ω, respectively. As shown in Fig. 8b,c, the N115 membrane exhibited the poorest catalytic performance on conversing CO_2_ to CO. Between 1.6 and 2.6 V, the COCE and the *j*_*CO*_ obtained with the N115 membrane was less than 40% and 10 mA cm^−2^, respectively. And the COEE obtained with the N115 membrane was the lowest among the four membranes (see Fig. 8d). This phenomenon was consistent with Kutz et al*.*^15^, that the anion exchange membrane (AEM) exhibited better catalytic performance in the conversion of CO_2_ to CO. With the use of AEM, the H^+^ will not be transported to the cathode, so the hydrogen evolution reaction was suppressed^9^. Among the three AEMs, the G60 membrane exhibited excellent catalytic performance. Under the same cell potential (from 1.8 to 2.6 V), the total current density obtained with the G60 membrane was almost twice that of the FAA50 membrane. Using the G60 membrane, the *j*_*CO*_ achieved 149.6 mA cm^−2^ at 2.0 V, with high current efficiency (95.0%) and energy efficiency (69.7%) in the conversion of CO_2_ to CO. This result was not surprising as the G60 membrane was developed especially for CO_2_ electrolysis^15^, and has been commercialized and adopted by more and more researchers^38–40^.

Combined with the EIS results in Figure S10, the total current density obtained with a smaller Rs was increased, which was the same as the previous test results under different configurations. According to the corresponding technical datasheet, the area resistance of the PK75 membrane (1.2–2.0 Ω cm^−2^ in Cl^−^ form) is larger than that of the FAA50 membrane (0.6–1.5 Ω cm^−2^ in Cl^−^ form), and the FAA50 membrane (45–55 µm) is thinner than the PK75 membrane (70–80 µm). This may explain why the Rs measured with the two membranes were nearly four times different. The best-performance G60 membrane is not only thin (50 µm) but also has the lowest average area resistance (0.045 Ω cm^−2^) under alkaline conditions.

Besides, hydrogen evolution reaction was more likely to occur when using N115 membrane, and so did the formate product. As shown in Figure S13 and S14, the current efficiency of formate obtained with N115 membrane was 23.6% at 2.2 V. No formate accumulation was observed when using anion exchange membranes. As shown in Figure S15, the formate concentration decreased with sample collection and deionized water replenishment.

## Discussion

In this work, beyond the properties of the catalyst, we extensively explored the influence of many other factors on the selectivity of CO production in continuous CO_2_ electrolysers. Compared with the similar structure and operation mode of the H-cell, adding a thin liquid buffer layer between the cathode and the membrane can improve the catalytic performance by promoting the diffusion of CO_2_ gas to the catalyst surface. A compact gas-phase MEA structure CO_2_ electrolyser was preferred, which has lower Rs and excellent CO_2_ gas mass transfer. PTFE was more suitable than Nafion as a binder for CO_2_RR GDE preparation. When the mass ratio of total-catalyst-to-PTFE was 7:1, the total current density reached 131.0 mA cm^−2^ at a low cell potential of 2.0 V, and the current efficiency and energy efficiency of CO production were 93.72% and 68.7%, respectively. Through the test and comparison of four kinds of membranes, it was found that the anion exchange membrane exhibited higher current efficiency and partial current density in the conversion of CO_2_ to CO. Choosing the right membrane for different target products, then twice as much can be accomplished with half the effort. With the development of CO_2_ electrolysis technology to continuous electrolysers with high current density, our research may pave the way for other research groups to develop new catalysts in full-cell CO_2_ electrolysers. It is indeed of great interest to further study whether the conclusion obtained in this study is suitable for other kinds of catalysts.

## Methods

### Materials

KOH (≧85.0%), KHCO_3_(≧99.5%), urea (≧99.0%), NaBH_4_ (≧98.0%), ethanol (≧99.7%); HClO_4_(70.0–72.0%), and HAuCl_4_ (≧99.9%) was purchased from Shanghai Lingfeng and Shanghai Chemical Reagent Co., Ltd respectively; Nafion solution (5 wt%) and polytetrafluoroethylene dispersion (Teflon PTFE DISP 30LX) was purchased from Dupont; anion exchange membrane (Fumasep FAA-3-50), carbon black (Vulcan XC 72R) and carbon paper (Toray, TGP-H-60) was purchased via Fuel Cell Store website. All other chemicals were purchased from Sinopharm Chemical Reagent Co., Ltd. Milli-Q ultrapure water (Millipore, ≥ 18 MΩ∙cm) was used throughout the work.

### Characterizations

The morphologies and the phase identification of the Au/CN catalysts were examined by transmission electron microscopy (TEM, Hitachi HT7700) and X-ray diffraction (Haoyuan, DX-27mini) respectively. The X-ray photoelectron spectroscopy (Thermo ESCALAB 250XI) measurement was performed. The actual mass ratio of gold in the catalyst was determined by an inductively coupled plasma emission spectrometer (ICP, Optima8300DV). The morphologies of the spray-prepared cathode were analyzed by scanning electron microscopy (SEM, Hitachi TM3030). The hydrophobic and hydrophilic performance of the prepared electrodes were characterized by static contact angles (Dataphysics, JY-82B Kruss DSA100).

### Catalysts and gas diffusion electrodes preparation

The Au nanoparticles supported on N-doped carbon (Au/CN) were synthesized by following our previous report^7^. The gold mass ratio of the Au/CN catalysts was 20 wt% and the particle size of gold nanoparticles was mainly distributed around 2 nm. Hydrophobic carbon paper (Toray, TGP-H-60) was used as the substrate, and no microporous layer was constructed. The Au/CN catalyst was dispersed in a solvent comprised of 1:1 (volume ratio) ethanol and water. After 20-min ultrasonication, 4 mg mL^−1^ (calculated based on the total mass of catalyst) ink was obtained. For experiments on different cathode feeding methods and electrolyser structures, Nafion dispersion was used as the binder, the mass ratio of total-catalyst-to-binder was 3:1. To investigate the influence of binder on CO_2_RR performance, different amounts of Nafion or PTFE dispersion (the mass ratios of total-catalyst-to-binder were 3:1, 5:1, 7:1, 10:1, respectively) were added. The total mass of catalyst loading of the gas diffusion electrode was 1.0 ± 0.1 mg cm^−2^. As-prepared electrodes need to be sintered for one hour before use, Nafion 130 °C and PTFE binders 330 °C, respectively. Nickel foam (1 × 1 cm^2^) was used as an anode catalyst to facilitate oxygen evolution. For three-electrode tests, 6.3 μL ink was dropped on the glassy carbon electrode with a diameter of 4 mm and dried at room temperature, making a mass loading of 0.2 mg cm^−2^ (calculated based on the total mass of catalyst).

### Full-cell tests

For the MEA structure, the humidified CO_2_ or CO_2_-saturated 0.5 M KHCO_3_ was induced into the cathode chamber, while 2 M KOH was circulated in the anode chamber. Under the three operation conditions (Figure S3), the flow rates of gas and liquid were set to 15 sccm and 30 ml min^−1^, respectively. Four different polymer electrolyte membranes, Fumasep FAA-3-PK-75, Nafion 115, Fumasep FAA-3-50, and Sustainion X37-50 Grade 60 were used in this research. For ease of illustration, they are referred to as PK75, N115, FAA50, and G60, respectively. Except for the membrane-related tests, the Fumasep FAA-3-50 membrane was used in other tests.

At each given cell voltage (1.6 V, 1.8 V, 2.0 V, 2.2 V, 2.4 V, and 2.6 V), a 20-min electrolytic test was performed using an electrochemical workstation (IVIUM, CompactStat.h A32718). The export gas was passed into deionized water to absorb liquid phase product. The actual gas outlet flow rate was monitored by a mass flow meter (Sevenstar, D07), 1 mL of the dried effluent gas was sampled automatically into a gas chromatograph (GC-2030) every ten minutes. At the end of electrolysis under each given cell voltage, 3 mL water absorption liquid was extracted and 3 mL deionized water was replenished. Formate concentration was examined by UV spectrophotometer (Metash, UV-5800H). At least two fresh-made parallel electrodes were tested for each sample. The electrochemical impedance spectroscopy (EIS) measurements were conducted from 100 kHz to 1 Hz with 10 mV amplitude under open circuit potential. There was no reference electrode included in the flow cell and no *iR* compensation was made unless otherwise specified. All the experiments were carried out at room temperature (25 °C) and ambient pressure.

### Electrochemical measurements

For the three-electrode system tests, a Pt foil and saturated calomel electrode (SCE) were used as the counter electrode and reference electrode respectively. Linear sweep voltammetry (LSV) scans were performed at 1 mV s^−1^ in 0.5 M KHCO_3_ saturated with CO_2_ (pH 7.3). The ECSA of the Au catalyst was calculated by the reduction peak area measured in 0.1 M HClO_4_. And 390 µC cm^−2^ was used as the reference charge value for Au^41^.

The current efficiency (CE) of a specific product is defined by the ratio of charge consumed in forming the product to the total charge consumed. The energy efficiency (EE) is defined by the ratio between the thermodynamic voltage to the practical cell voltage. The energy efficiency of CO (COCE) needs to be multiplied by its current efficiency^7^.

## Supplementary Information

Supplementary Information.
