# Supplementary material for: A study on improving the current density performances of CO2 electrolysers
Source: Sci Rep. 2021 May 27;11:11136. doi: 10.1038/s41598-021-90581-0 (PMC8159929; doi:10.1038/s41598-021-90581-0)
Supplement: Supplementary file 1 — Supplementary Information. [file 41598_2021_90581_MOESM1_ESM.docx]

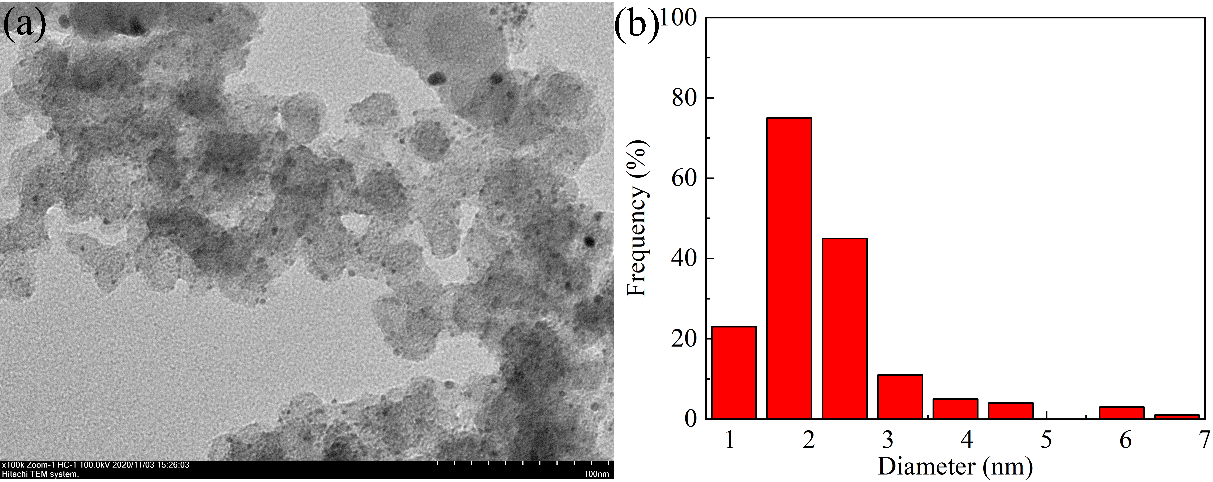


**Figure S1.** (a) TEM image of the Au/CN catalyst and (b) Histograms of Au nanoparticle size distribution extracted from TEM image.


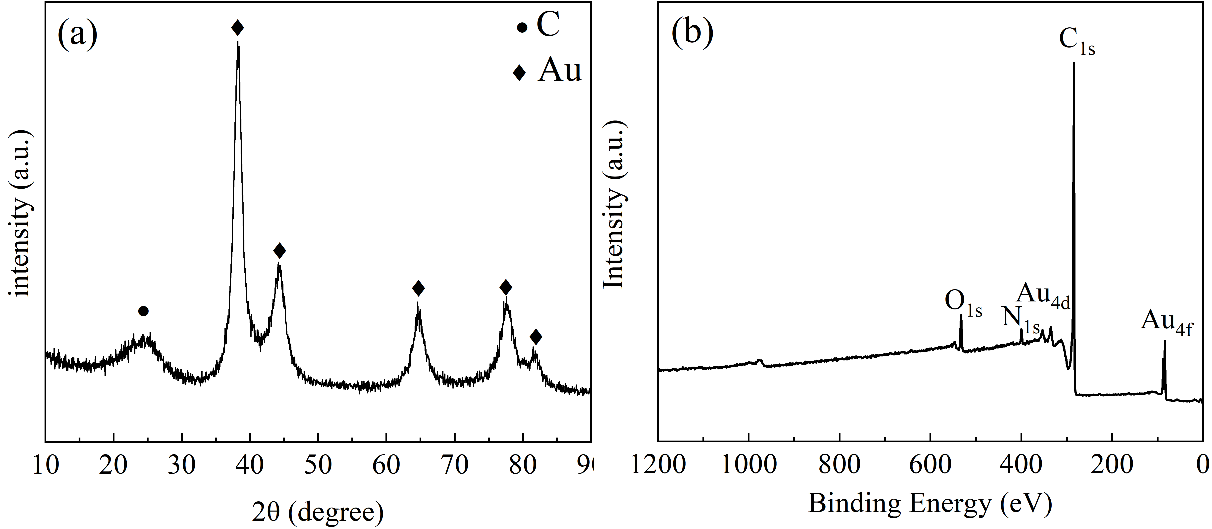


**Figure S2.** (a) XRD spectra and (b) XPS survey of the Au/CN catalyst.


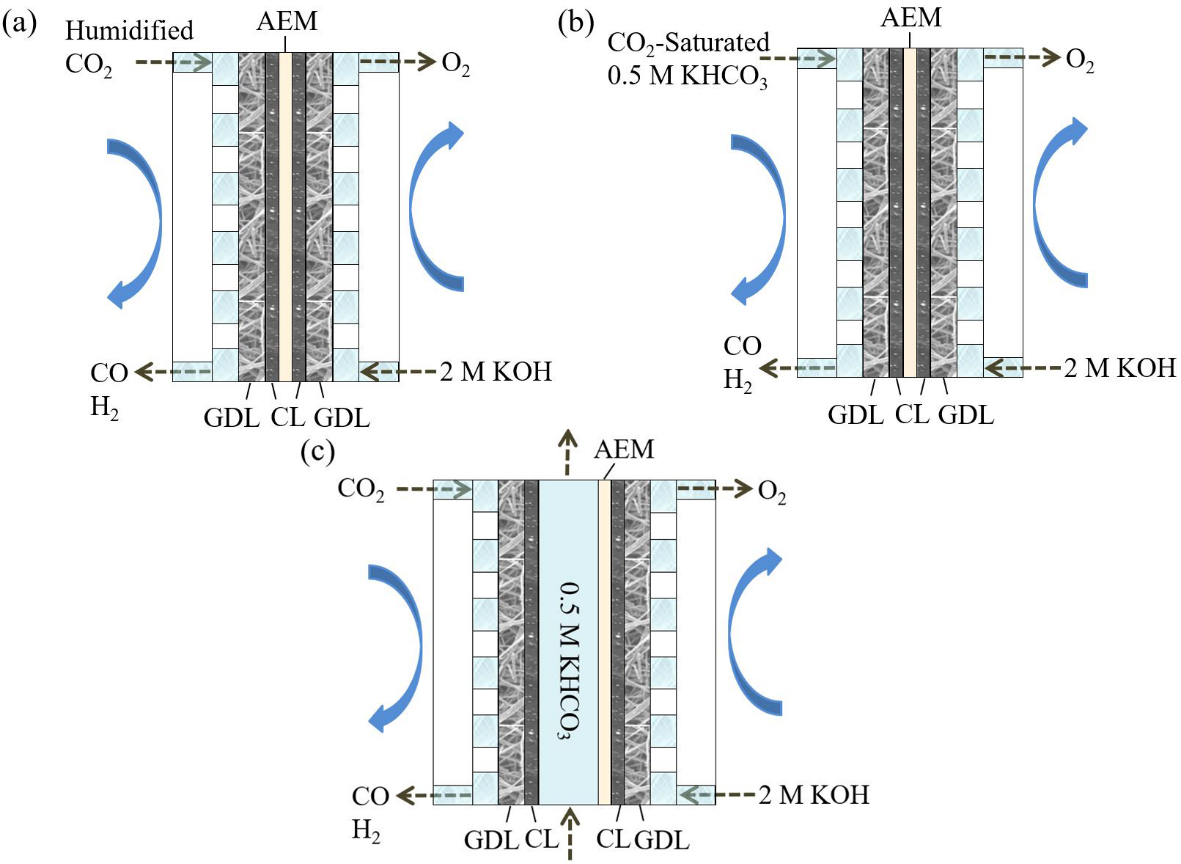


**Figure S3.** CO_2_ electrolyser configurations: (a) gas phase at the cathode, (b) CO_2_-saturated 0.5 M KHCO_3_ at the cathode, (c) gas phase at the cathode with a liquid buffer layer.


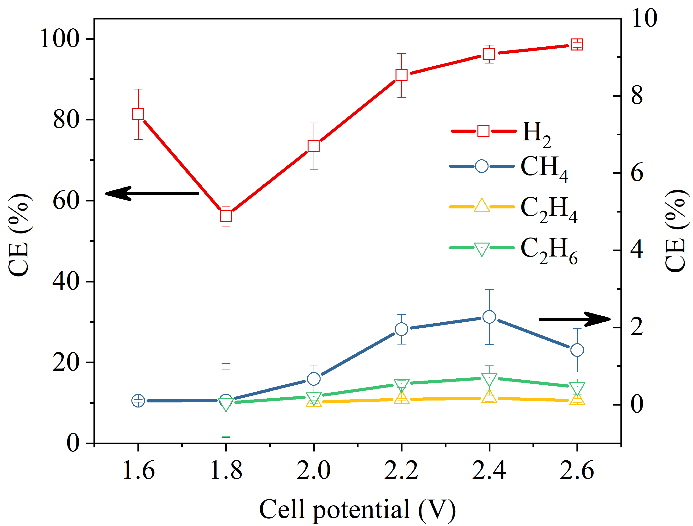


**Figure S4.** Current efficiencies of other gas products obtained with CO_2_-saturated 0.5 M KHCO_3_ as catholyte, as a function of the cell potential.


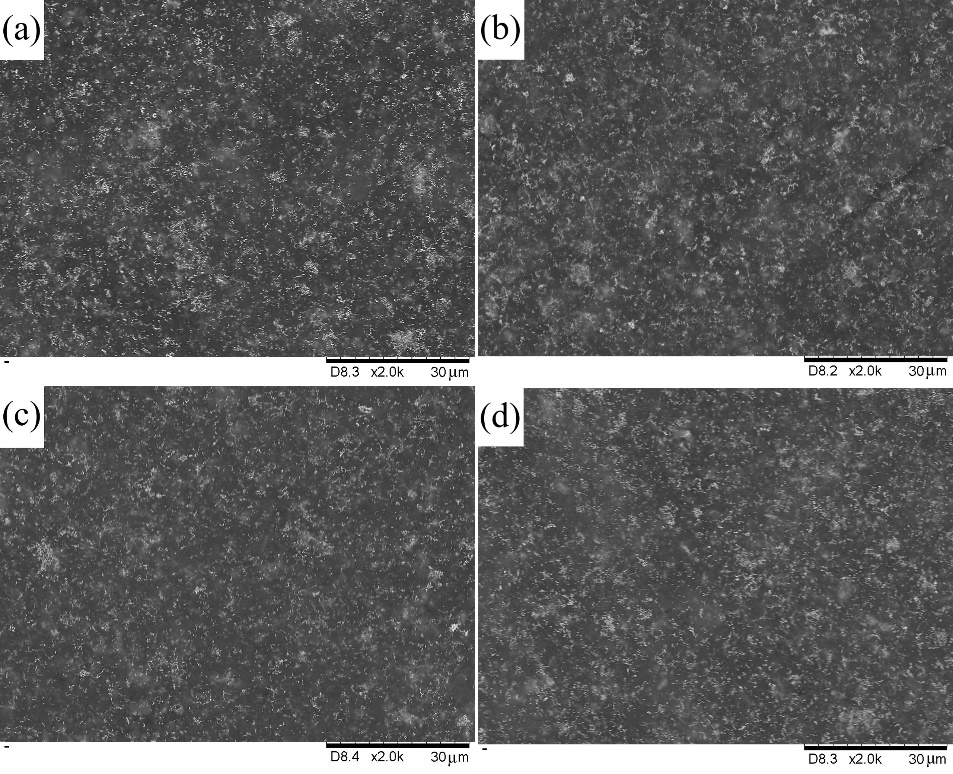


**Figure S5.** SEM images of gas diffusion electrodes with different PTFE additions (a) 3:1, (b) 5:1, (c) 7:1, (d) 10:1.


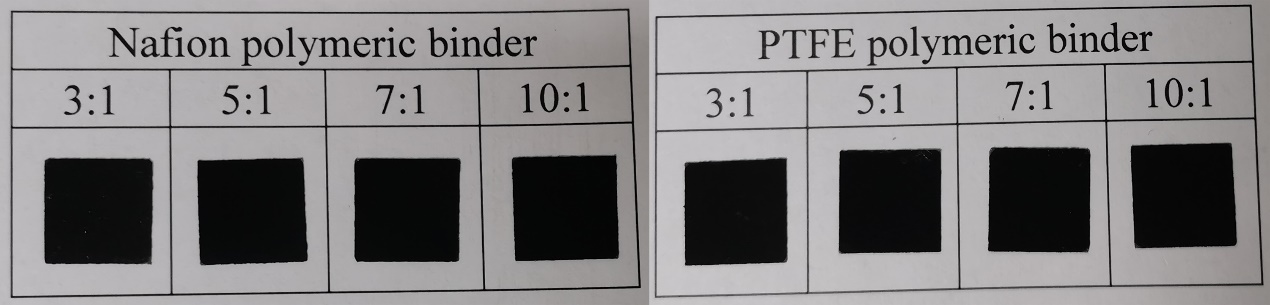


**Figure S6.** Digital photos of the air-sprayed electrodes with different polymeric binders.





**Figure S7.** Total current density as a function of time under different cell potential with 7:1 PTFE addition.


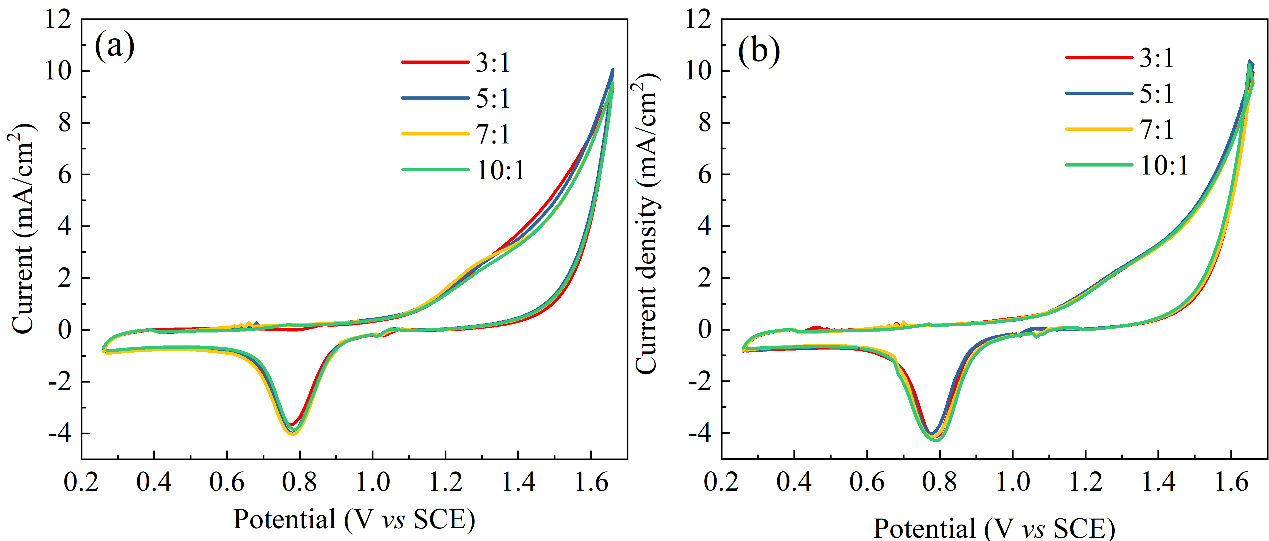


**Figure S8.** CV scans of Au/CN catalysts with different (a) Nafion and (b) PTFE contents in 0.1 M HClO_4_ at 50 mV/s scan rate.


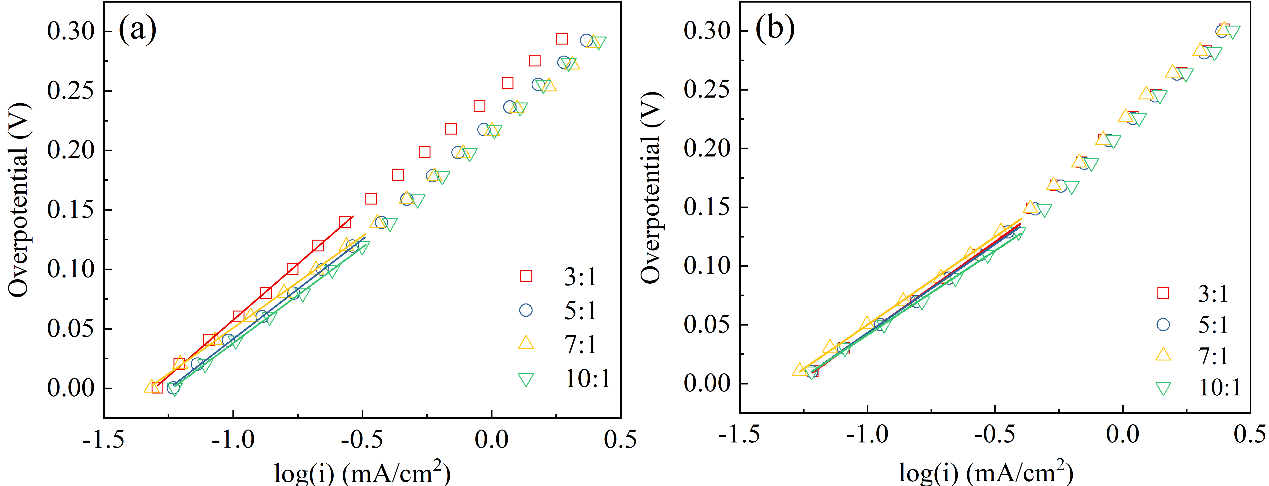


**Figure S9.** Tafel plots with different ionomer contents. (a) Nafion and (b) PTFE ionomer.

**Table S1.** The contact angles of the air-brushed electrodes with different ionomer contents, and the corresponding Tafel slope, i_0_ and ECSA of gold obtained on glass carbon electrode.

| Polymeric binder | Catalyst and ionomer mass ratio | Contact angle (^o^) | ECSA of gold (cm^2^) | Tafel slope (mV/dec) | i_0_ (*10^-5^ mA/cm^2^) |
| --- | --- | --- | --- | --- | --- |
| Nafion | 3:1 | 140.2 | 3.56 | 187 | 4.95 |
|  | 5:1 | 138.8 | 3.61 | 165 | 5.62 |
|  | 7:1 | 138.0 | 3.58 | 152 | 4.65 |
|  | 10:1 | 114.4 | 3.55 | 159 | 5.79 |
| PTFE | 3:1 | 140.6 | 3.57 | 155 | 5.31 |
|  | 5:1 | 139.8 | 3.59 | 151 | 5.20 |
|  | 7:1 | 138.9 | 3.60 | 150 | 4.64 |
|  | 10:1 | 118.0 | 3.58 | 143 | 5.15 |


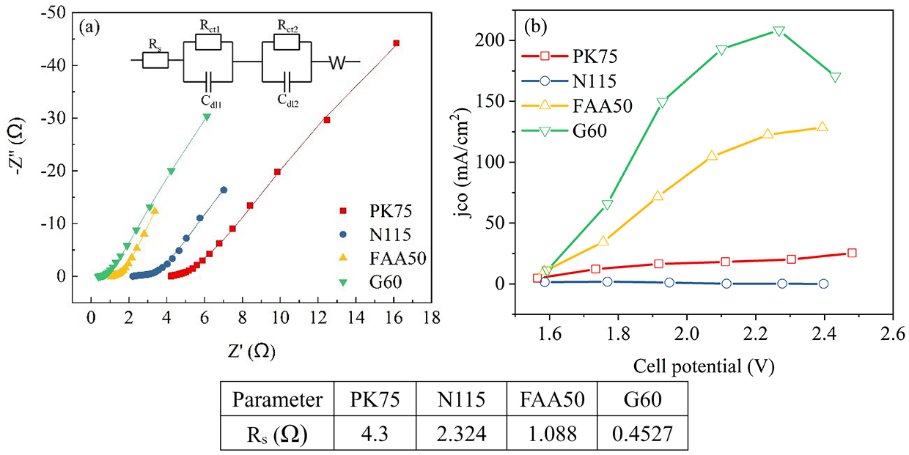


**Figure S10.** EIS and the relevant equivalent circuit acquired with different membrane in CO_2_ electrolyser under open-circuit level. Points and lines represent measured and fitted results, respectively. (b) j_CO_ as a function of the cell potential (after iR compensation).


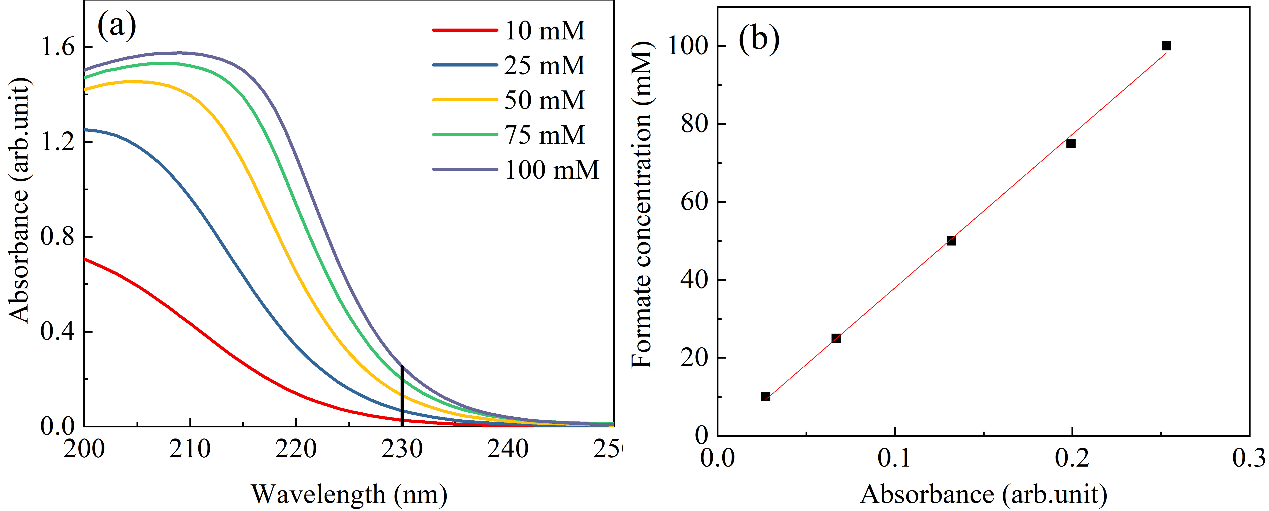


**Figure S11. (a)** UV-visible spectra for various concentrations of potassium formate solutions, (b) the concentration of potassium formate vs absorbance at 230 nm wavelength.


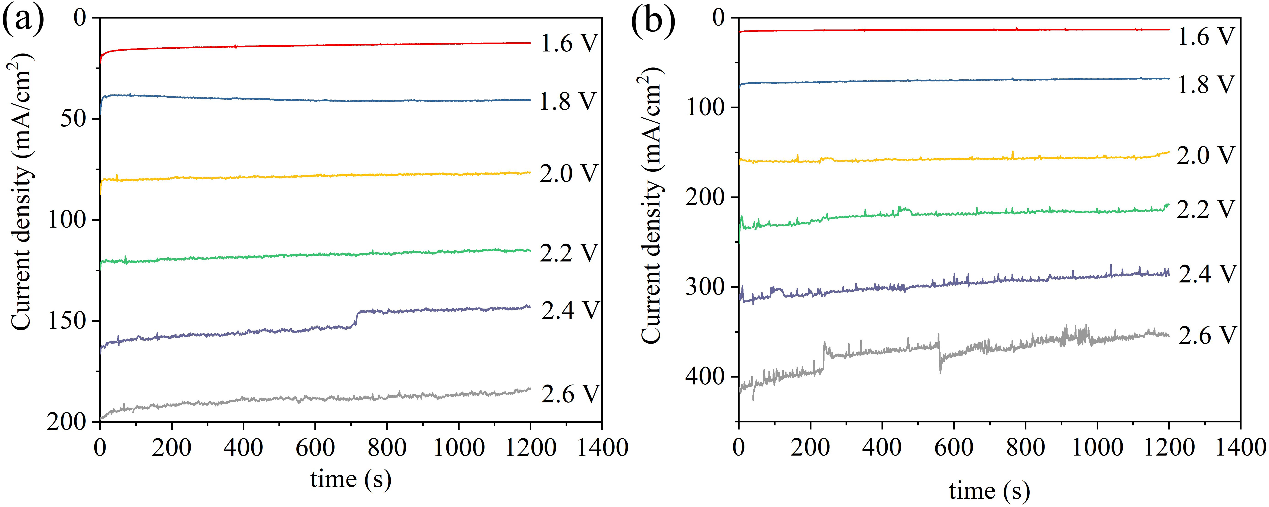


**Figure S12.** Total current density as a function of time under different cell potential with different membranes. (a) Fumasep^Ⓡ^ FAA-3-50, (b) Sustainion^TM^ X37-50 Grade 60.


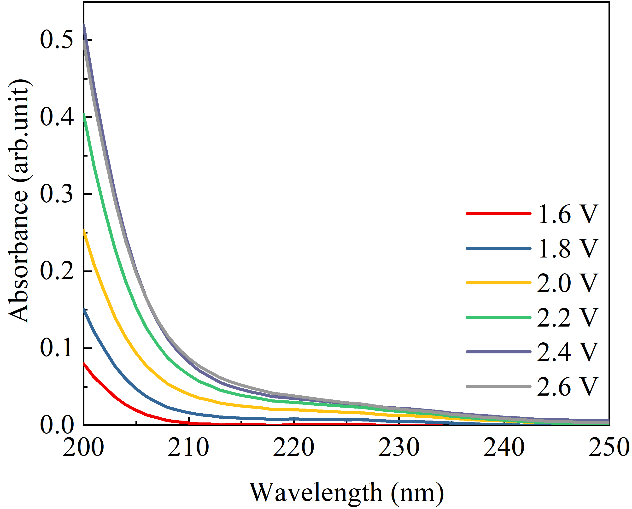


**Figure S13.** UV-visible spectra of water adsorption solution in cathode under different cell potential acquired with Nafion N115 membrane.


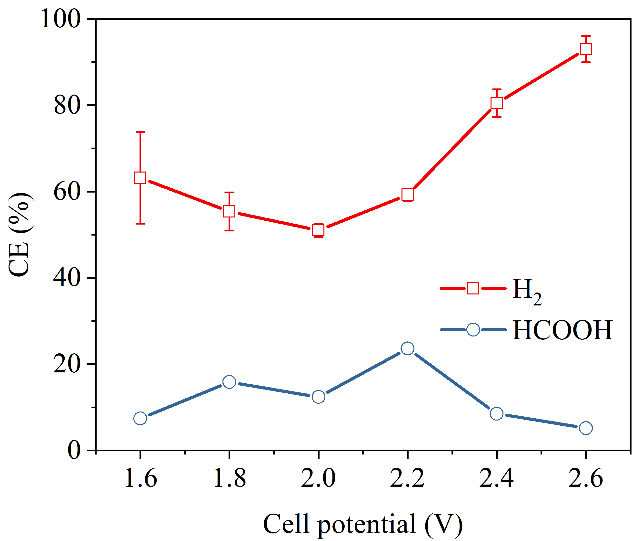


**Figure S14.** Current efficiencies of hydrogen and formate obtained with Nafion N115 membrane, as a function of the cell potential.


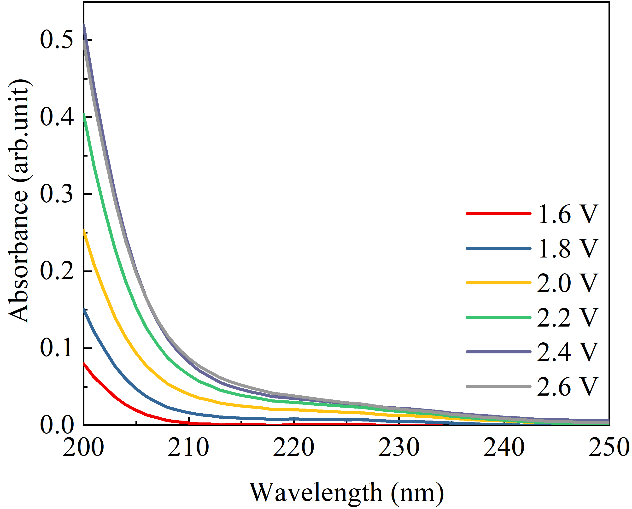


**Figure S15.** UV-visible spectra of water adsorption solution in cathode under different cell potential acquired with Fumasep^Ⓡ^ FAA-3-50 membrane.
